# Relationship among three common hematological premalignant conditions

**DOI:** 10.1038/s41375-023-01914-z

**Published:** 2023-05-05

**Authors:** Nicholas J. Boddicker, Sameer A. Parikh, Aaron D. Norman, Kari G. Rabe, Rosalie Griffin, Timothy G. Call, Dennis P. Robinson, Janet E. Olson, Angela Dispenzieri, Vincent Rajkumar, Shaji Kumar, Neil E. Kay, Curtis A. Hanson, James R. Cerhan, David Murray, Esteban Braggio, Tait D. Shanafelt, Celine M. Vachon, Susan L. Slager

**Affiliations:** 1 Division of Computational Biology, Mayo Clinic, Rochester, MN, USA.; 2 Division of Hematology, Mayo Clinic, Rochester, MN, USA.; 3 Division of Epidemiology, Mayo Clinic, Rochester, MN, USA.; 4 Division of Clinical Trials and Biostatistics, Mayo Clinic, Rochester, MN, USA.; 5 Department of Immunology, Mayo Clinic, Rochester, MN, USA.; 6 Department of Laboratory Medicine and Pathology, Mayo Clinic, Rochester, MN, USA.; 7 Department of Hematology and Oncology, Mayo Clinic, Phoenix, AZ, USA.; 8 Department of Medicine, Division of Hematology, Stanford University, Stanford, CA, USA.; 9 These authors contributed equally: Celine M. Vachon, Susan L. Slager.

Monoclonal B-cell lymphocytosis (MBL), monoclonal gammopathy of undetermined significance (MGUS), and clonal hematopoiesis (CH) are all asymptomatic hematological conditions characterized by clonal expansion of blood cells [1, 2]. These conditions are notably associated with increased risk of hematologic cancers. Each condition has an annual progression rate of ~1–2%/year, with MBL progressing to chronic lymphocytic leukemia (CLL) [3] or lymphoid malignancies [4], MGUS progressing mostly to multiple myeloma (MM) [5], and CH progressing predominately to myeloid neoplasms [2]. Additionally, these premalignant conditions (or a particular subtype of them) are associated with adverse outcomes, including increased risk of infections [6–8] and reduced overall survival [2, 4, 5]. Known risk factors for these conditions are limited but include aging [4, 9] and common inherited variants [10–12].

The prevalence of each of these conditions has been established, ranging from 5–25% overall, with particular subtypes having varying prevalence rates [2, 4, 9]. However, little is known about their co-occurrence. One hospital-based cohort of ~1500 non-hematological patients screened for MBL and MGUS found no significant association between them [13]. Another study of 777 subjects enrolled in the Monzino 80-plus cohort found no significant association between MGUS and CH [14]. Herein, we investigate the co-occurrence of MBL with MGUS and with CH, along with the associations between them and the prevalence of all three conditions.

This study was approved by the institutional review boards of Mayo Clinic and Olmsted Medical Center, and participants provided written informed consent. Study participants were a cross-sectional sample from the Mayo Clinic Biobank who were recruited between 2009–2016 from general medical practice clinics, randomly selected from participants who had no prior history of hematological cancers, 40 years or older, and provided a biospecimen needed for MBL and MGUS screening [4]. For a more representative cohort, we included participants who resided predominantly from counties surrounding Mayo Clinic and therefore who typically receive their general medical care at Mayo Clinic. CH screening was conducted on a subset of these individuals and were selected based on MBL status (a case-control study comprised of 330 individuals with MBL and 588 age- and sex-matched individuals without MBL).

The methods used for MBL screening [4, 7] and MGUS screening [9] have been previously published. For CH, peripheral blood DNA was used to sequence the coding regions from 42 CH-related genes (Supplementary Table 1) using the Illumina HiSeq 4000. The average coverage depth was >1000x. Somatic mutations were called using MuTect2. Variants were excluded if the number of reads supporting variant alleles was <8, variant allele fraction (VAF) was <0.01, gnomAD allele frequency was ≥0.005, more than 80% of the variant reads came from a single strand, or the VAF did not deviate from 50% at a *p* value of at least 0.00001 unless reported in COSMIC at least 10 times. Loss-of-function variants (nonsense, frameshift, and consensus splice sites) and missense variants, classified as pathogenic or likely pathogenic, were used to identify individuals with CH. Different laboratory technicians performed each premalignant screening assay, thus were blinded to the results of the other screening assays.

Logistic regression was used to estimate odds ratios (OR) and 95% confidence intervals (CI) using R (version 3.6.2). All analyses were adjusted for age and sex; in addition, association analyses between CH and MGUS were also adjusted for MBL status.

## RESULTS/DISCUSSION

In total, 1630 individuals were screened for MBL and MGUS; 1151 (70.6%) were negative for both, 275 (16.9%) identified to have MBL only, 147 (9.0%) identified to have MGUS only, and 57 (3.5%) identified to have both MBL and MGUS (Table 1). Individuals with MBL only were, on average, older than those without MBL/MGUS (median 70 vs. 63 years of age, respectively, *p* < 0.001) and were more likely to be male (54.5% vs. 41.0%, respectively, *p* < 0.001). CLL-like MBL was the most common MBL subtype (87.3%), followed by non-CLL-like (8.4%), and atypical MBL (4.4%). Individuals with MGUS only were also older than those without MBL/MGUS (median 68 vs. 63 years of age, respectively, *p* < 0.001); however, there was no significant difference in sex (*p* = 0.13). Non-IgM (86.0%), and particularly IgG MGUS (63.5%), were the most common MGUS isotypes.

Among the 57 individuals with both MGUS and MBL, 49.1% were male and median age was 72 years (Table 1). The frequency of MBL and MGUS coexisting was 3.5%, which is higher than the 0.4% reported previously [13]. This discrepancy is likely due to the higher sensitivity of MGUS detection used in the current study [15]. When restricted to the detection metrics of M-spike >0.2 g/dl, the frequency of those with MBL and MGUS was similar to the prior report (0.8%), although the prior publication was unclear of their M-spike threshold.

A subset of 918 individuals were sequenced for CH, of whom 249 (27.1%) had CH detected. Genes *DNMT3A*, *TET2*, and *ASXL1* (DTA-CH) made up 65.1% of the CH carriers (Table 1).

Although CH screening was not available on the entire cohort, it was complete for those with both MBL and MGUS (Table 1), allowing for accurate estimates of all three conditions. In total, 17 (1.0%) individuals concurrently had all three premalignant conditions. These individuals were 47% male with a median age of 78 years. CLL-like MBL was the most common MBL subtype (47.1%) and non-IgM MGUS were the most common MGUS isotypes (73.7%).

Further, CH screening was available on all but 2 individuals with MBL from the full cohort, allowing for accurate estimate of the prevalence of CH and MBL. In total, 92 (5.6%) out of the1,630 individuals concurrently had MBL and CH. We did not have sequencing completed on all MGUS in the full sample and thus were unable to estimate their co-occurrence.

**Fig. 1:**
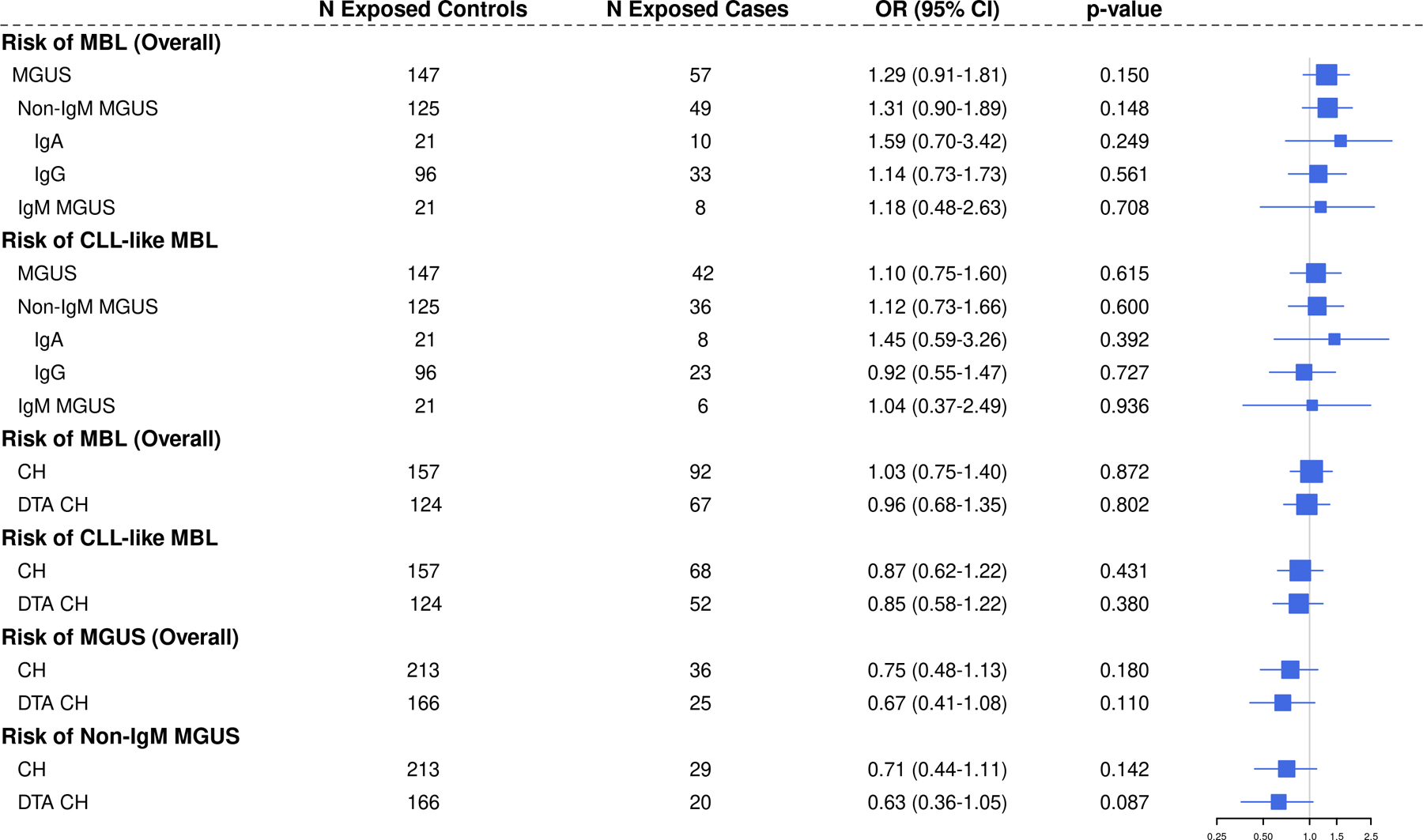
Forest plot of associations between premalignant conditions of monoclonal B-cell lymphocytosis (MBL), monoclonal gammopathy of undetermined significance (MGUS), and clonal hematopoiesis (CH). DTA CH: *DNMT3A*, *TET2*, or *ASXL1* somatic mutation carrier.

The associations among these premalignant conditions are shown in Fig. 1. There was an elevated OR for the association of MGUS overall with MBL (OR = 1.29, 95% CI: 0.91–1.81, *p* = 0.15) which did not reach statistical significance. The OR was highest for IgA MGUS 1.59 (95% CI: 0.70–3.42), but small sample sizes limited power for subtype specific associations.

When we subset to individuals with CLL-like MBL, there was no evidence of an association between MGUS overall and CLL-like MBL (OR = 1.10, 95% CI: 0.75–1.60, *p* = 0.62, Fig. 1). Similarly, there was no evidence of an association between CLL-like MBL and non-IgM MGUS or any of the MGUS isotypes, although power was limited for these MGUS isotype analyses. Moreover, there was no statistical evidence of an association between CH and MBL overall or with CLL-like MBL (ORs 1.03 and 0.87, respectively). There was also no evidence of an association between DTA-CH and MBL overall or CLL-like MBL (ORs 0.96 and 0.85, respectively). When investigating the relationship between CH and MGUS, we found no statistical evidence of an association, although there may be an inverse relationship between CH and MGUS (Fig. 1), like the nonsignificant association in Da Via et al. [14]. The results were similar when analysis was limited to individuals with CH who had a VAF > 2% (Supplementary Table 2) [2].

Lastly, there are 19 genes in our CH gene list (Supplementary Table 1) that are also recurrently mutated in lymphoid malignancies. Because MBL and MGUS are precursors to lymphoid malignancies, we restricted our CH definition to exclude these 19 genes and found similar results to our overall analysis (Supplementary Table 2).

In summary, this study of 1630 individuals found no statistical evidence of a relationship among risk of common hematological premalignant conditions. Particularly, we found no evidence of associations between MGUS with CLL-like MBL or between CH with CLL-like MBL. However, further studies are needed to evaluate associations among the rare subtype conditions due to limited sample size herein. Overall, these results suggest these premalignant conditions do not appear to cluster together. Future studies are needed to investigate whether those with two or more of these conditions have increased clinical phenotypes (e.g., cytopenias, cancers, infections) or mortality compared to those with only one or no premalignant condition.

## Supplementary Material

Supplemental

## Figures and Tables

**Table 1.** Patient characteristics by premalignant condition status.

	No MBL/MGUS (*N* = 1151)	MBL only (*N* = 275)	MGUS only (*N* = 147)	Both MBL/MGUS (*N* = 57)	Total (*N* = 1630)
Sex					
Female	679 (59.0%)	125 (45.5%)	77 (52.4%)	29 (50.9%)	910 (55.8%)
Male	472 (41.0%)	150 (54.5%)	70 (47.6%)	28 (49.1%)	720 (44.2%)
Age at MBL screening (years)					
Median	63.0	70.0	68.0	72.0	65.0
Range	40.0–89.0	43.0–96.0	43.0–94.0	53.0–94.0	40.0–96.0
MBL immunophenotype					
Atypical	-	12 (4.4%)	-	5 (8.8%)	17 (5.1%)
CLL-like	-	240 (87.3%)	-	42 (73.7%)	282 (84.9%)
Non-CLL-like	-	23 (8.4%)	_-_	10 (17.5%)	33 (9.9%)
MBL clonal size					
HC-MBL	-	15 (5.5%)	_-_	7 (12.3%)	22 (6.6%)
LC-MBL	-	260 (94.5%)	-	50 (87.7%)	310 (93.4%)
MGUS heavy chain isotype					
Missing	-	-	1	0	1427
Biclonal	-	-	8 (5.5%)	6 (10.5%)	14 (6.9%)
IgA	-	-	21 (14.4%)	10 (17.5%)	31 (15.3%)
IgG	_-_	_-_	96 (65.8%)	33 (57.9%)	129 (63.5%)
IgM	_-_	_-_	21 (14.4%)	8 (14.0%)	29 (14.3%)
M-Spike (g/dl)					
Missing	_-_	_-_	13	_-_	_-_
<0.2	-	-	103 (75.2%)	41 (74.5%)	144 (75.0%)
0.2–1.5	-	-	29 (21.2%)	13 (23.6%)	42 (21.9%)
≥1.5	-	-	5 (3.6%)	1 (1.8%)	6 (3.1%)
CH (overall)^a^					
Missing	644	2	66	0	712
No	369 (72.8%)	198 (72.5%)	62 (76.5%)	40 (70.2%)	669 (72.9%)
Yes	138 (27.2%)	75 (27.5%)	19 (23.5%)	17 (29.8%)	249 (27.1%)
*DNMT3A* CH	63 (12.4%)	37 (13.6%)	5 (6.2%)	4 (7.0%)	109 (11.9%)
*TET2* CH	45 (8.9%)	18 (6.6%)	10 (12.3%)	4 (7.0%)	77 (8.4%)
*ASXL1* CH	10 (2.0%)	4 (1.5%)	1 (1.2%)	3 (5.3%)	18 (2.0%)
nonDTA CH	41 (8.1%)	28 (10.3%)	8 (9.9%)	10 (17.5%)	87 (9.5%)

*MBL* monoclonal B-cell lymphocytosis, *MGUS* monoclonal gammopathy of undetermined significance, *CH* clonal hematopoiesis, *LC-MBL* low-count *MBL*, *HC-MBL* high-count *MBL*, *nonDTA* non *DNMT3A*, *TET2*, or *ASXL1* somatic mutation carrier.

a Frequencies exclude individuals missing CH sequencing information.

## Data Availability

The data that support the findings of this study are available from the corresponding author upon reasonable request.
