# Supplementary material for: Relationship among three common hematological premalignant conditions
Source: Leukemia. Author manuscript; Available in PMC 2023 Sep 27. (PMC10400408; doi:10.1038/s41375-023-01914-z)
Supplement: Supplemental [file NIHMS1915966-supplement-Supplemental.pdf]

Supplemental Table 1. Genes examined for somatic variants in the targeted sequencing to identify individuals with clonal hematopoiesis

**Gene Name**
*ASXL1**
ASXL2
*ATM**
*BCOR**
BCORL1
*BIRC3**
*BRAF**
*BRCC3**
*CARD11**
CBL
CEBPA
*CREBBP**
CUX1
DNMT3A
GNAS
*GNB1**
IDH1
IDH2
JAK2
KDM6A
*KMT2D**
*KRAS**
*MYD88**
NF1
*NOTCH1**
NOTCH2
*NRAS**
PPM1D
*PTPN11**
RAD21
RUNX1
SETBP1
*SETD2**
*SF3B1**
SRSF2
STAT3
*SYNE1**
TET2
*TP53**
U2AF1
WT1
ZRSR2

* Genes associated with both clonal hematopoiesis and lymphoid malignancies.

Supplemental Table 2. Association between clonal hematopoiesis (CH) and monoclonal B-cell lymphocytosis (MBL) or monoclonal gammopathy of undetermined significance (MGUS) for subset analyses of CH variants with a variant allele fraction (VAF) of ≥2% and CH restricted to myeloid associated genes

	CH VAF ≥ 2%					Myeloid-CH* Only Genes				
	N Exposed Controls	N Exposed Cases	Odds Ratio	95% CI	p-value	N Exposed Controls	N Exposed Cases	Odds Ratio	95% CI	p-value
**Risk of MBL (Overall)**										
CH	107	71	1.20	0.85-1.69	0.304	140	78	0.96	0.69-1.33	0.811
DTA CH	81	49	1.10	0.74-1.62	0.646	117	62	0.92	0.65-1.30	0.647
**Risk of CLL-like MBL**										
CHIP	107	51	1.00	0.68-1.45	0.998	140	60	0.87	0.61-1.23	0.427
DTA CH	81	38	0.98	0.64-1.49	0.932	117	49	0.85	0.58-1.23	0.394
**Risk of MGUS (Overall)**										
CH	152	26	0.75	0.45-1.19	0.227	189	29	0.69	0.43-1.08	0.112
DTA CH	114	16	0.61	0.33-1.06	0.094	157	22	0.65	0.38-1.05	0.086
**Risk of Non-IgM MGUS**										
CH	152	20	0.67	0.38-1.11	0.132	189	23	0.64	0.38-1.04	0.083
DTA CH	114	13	0.57	0.29-1.04	0.081	157	17	0.58	0.32-0.99	0.057

* Gene excluded from Myeloid-CH definition because of they are also associated with lymphoid malignancies: *ASXL1, ATM, BCOR, BIRC3, BRAF, BRCC3, CARD11, CREBBP, GNB1, KMT2D, KRAS, MYD88, NOTCH1, NRAS, PTPN11, SETD2, SF3B1, SYNE1, TP53*
