# Supplementary material for: Relationship among three common hematological premalignant conditions
Source: Leukemia. 2023 May 5;37(8):1719–22. doi: 10.1038/s41375-023-01914-z (PMC10400408; doi:10.1038/s41375-023-01914-z)
Supplement: Supplementary file 1 [file 41375_2023_1914_MOESM1_ESM.docx]

**Supplemental Table 1.** Genes examined for somatic variants in the targeted sequencing to identify individuals with clonal hematopoiesis

| **Gene Name** |
| --- |
| *ASXL1** |
| *ASXL2* |
| *ATM** |
| *BCOR** |
| *BCORL1* |
| *BIRC3** |
| *BRAF** |
| *BRCC3** |
| *CARD11** |
| *CBL* |
| *CEBPA* |
| *CREBBP** |
| *CUX1* |
| *DNMT3A* |
| *GNAS* |
| *GNB1** |
| *IDH1* |
| *IDH2* |
| *JAK2* |
| *KDM6A* |
| *KMT2D** |
| *KRAS** |
| *MYD88** |
| *NF1* |
| *NOTCH1** |
| *NOTCH2* |
| *NRAS** |
| *PPM1D* |
| *PTPN11** |
| *RAD21* |
| *RUNX1* |
| *SETBP1* |
| *SETD2** |
| *SF3B1** |
| *SRSF2* |
| *STAT3* |
| *SYNE1** |
| *TET2* |
| *TP53** |
| *U2AF1* |
| *WT1* |
| *ZRSR2* |

* Genes associated with both clonal hematopoiesis and lymphoid malignancies.

**Supplemental Table 2. Association between clonal hematopoiesis (CH) and monoclonal B-cell lymphocytosis (MBL) or monoclonal gammopathy of undetermined significance (MGUS) for subset analyses of CH variants with a variant allele fraction (VAF) of ≥2% and CH restricted to myeloid associated genes**

|  | **CH VAF ≥ 2%** |  |  |  |  | **Myeloid-CH* Only Genes** |  |  |  |  |
| --- | --- | --- | --- | --- | --- | --- | --- | --- | --- | --- |
|  | **N Exposed Controls** | **N Exposed Cases** | **Odds Ratio** | **95% CI** | **p-value** | **N Exposed Controls** | **N Exposed Cases** | **Odds Ratio** | **95% CI** | **p-value** |
| **Risk of MBL (Overall)** |  |  |  |  |  |  |  |  |  |  |
| CH | 107 | 71 | 1.20 | 0.85-1.69 | 0.304 | 140 | 78 | 0.96 | 0.69-1.33 | 0.811 |
| DTA CH | 81 | 49 | 1.10 | 0.74-1.62 | 0.646 | 117 | 62 | 0.92 | 0.65-1.30 | 0.647 |
| **Risk of CLL-like MBL** |  |  |  |  |  |  |  |  |  |  |
| CHIP | 107 | 51 | 1.00 | 0.68-1.45 | 0.998 | 140 | 60 | 0.87 | 0.61-1.23 | 0.427 |
| DTA CH | 81 | 38 | 0.98 | 0.64-1.49 | 0.932 | 117 | 49 | 0.85 | 0.58-1.23 | 0.394 |
| **Risk of MGUS (Overall)** |  |  |  |  |  |  |  |  |  |  |
| CH | 152 | 26 | 0.75 | 0.45-1.19 | 0.227 | 189 | 29 | 0.69 | 0.43-1.08 | 0.112 |
| DTA CH | 114 | 16 | 0.61 | 0.33-1.06 | 0.094 | 157 | 22 | 0.65 | 0.38-1.05 | 0.086 |
| **Risk of Non-IgM MGUS** |  |  |  |  |  |  |  |  |  |  |
| CH | 152 | 20 | 0.67 | 0.38-1.11 | 0.132 | 189 | 23 | 0.64 | 0.38-1.04 | 0.083 |
| DTA CH | 114 | 13 | 0.57 | 0.29-1.04 | 0.081 | 157 | 17 | 0.58 | 0.32-0.99 | 0.057 |

* Gene excluded from Myeloid-CH definition because of they are also associated with lymphoid malignancies: *ASXL1, ATM, BCOR, BIRC3, BRAF, BRCC3, CARD11, CREBBP, GNB1, KMT2D, KRAS, MYD88, NOTCH1, NRAS, PTPN11, SETD2, SF3B1, SYNE1, TP53*
